# Chromosome‐level genome assembly of *Ctenoplusia agnata* and its potential application in Plusiinae pest management

**DOI:** 10.1002/ps.8949

**Published:** 2025-06-05

**Authors:** Juil Kim, Hossain Md Faruquee, Murtaza Khan

**Affiliations:** ^1^ Agriculture and Life Science Research Institute Kangwon National University Chuncheon Republic of Korea; ^2^ Department of Plant Medicine, Division of Bio‐Resource Sciences, College of Agriculture and Life Science Kangwon National University Chuncheon Republic of Korea

**Keywords:** *Ctenoplusia agnata*, genome assembly, Plusiinae, Noctuidae, Pest management

## Abstract

**BACKGROUND:**

*Ctenoplusia agnata* is a polyphagous pest affecting bean crops and cruciferous vegetables across East Asian countries, including Korea. Its periodic outbreaks cause significant damage to crops. The lack of a genome assembly has limited research on integrated pest management strategies and understanding its phylogenetic relationships with related species.

**RESULTS:**

We sequenced the genome of a Korean *C. agnata* using PacBio Revio long‐read combined with Pore‐C scaffolding. The final assembly (GCA_041146325.1, 406.7 Mb) comprises 70 scaffolds, with the largest 31 scaffolds representing 95.1% of the assembly, corresponding to the expected 31 chromosomes. BUSCO analysis confirmed high completeness with 98.8% gene coverage and 36% GC content. The assembly achieved a scaffold N50 of 13.2 Mb, scaffold L50 of 14, and 28.57% repeat sequence content. A total of 12 726 protein‐coding genes were predicted, of which 12 635 (99.28%) were functionally annotated. Comparative analysis with *Chrysodeixis includens* and *Trichoplusia ni*, two Plusiinae species, revealed that while *C. agnata* shares the chromosome number and broad synteny with *C. includens*; phylogenetic analyses place it closer to *T. ni*, suggesting distinct evolutionary trajectories despite structural conservation. Analysis of five major detoxification enzyme gene families (CYP, CCE, GST, UGT, and ABC) revealed that *C. agnata* contains fewer or similar detoxification genes compared to both *T. ni* and *C. includens*.

**CONCLUSION:**

The genome of *C. agnata* provides a valuable genetic resource for enhancing our understanding of the Plusiinae evolution and developing effective integrated pest management strategies. © 2025 The Author(s). *Pest Management Science* published by John Wiley & Sons Ltd on behalf of Society of Chemical Industry.

## Graphical abstract

Assembly of *Ctenoplusia agnata* genome at the chromosomal level and the use of pest management through comparative genomes with Plusiinae pests.

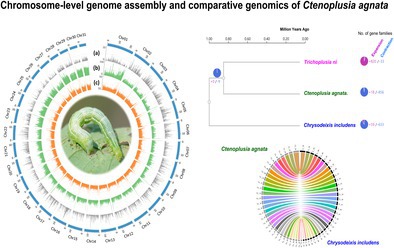

## 1 INTRODUCTION

The subfamily Plusiinae (family Noctuidae) is widely distributed across tropical, temperate, and arctic regions, with many species recognized as significant agricultural pests. *Ctenoplusia agnata*, a member of this subfamily, is a highly polyphagous lepidopteran pest that infests various economically important crops, including soybeans, tobacco, and cotton, leading to substantial agricultural losses.^1^, ^2^ Historical outbreaks of *C. agnata* in China, Korea, and Japan during the 1960s severely impacted soybean and vegetable yields, underscoring its potential as a major pest.^3^, ^4^

Two key ecological characteristics distinguish *C. agnata* as a formidable agricultural pest. First, its remarkable capacity for long‐distance migration enables rapid geographic spread and colonization of new agricultural areas, complicating monitoring and control efforts.^5^ Research has documented seasonal migration patterns covering hundreds of kilometers, allowing populations to exploit ephemeral resources and escape unfavorable conditions.^1^ Second, *C. agnata* exhibits significant cold tolerance, with physiological adaptations that permit overwintering in temperate regions and facilitate range expansion into previously unsuitable habitats.^6^ These combined traits enable *C. agnata* to persist across diverse agricultural landscapes and respond rapidly to changing environmental conditions, presenting unique challenges for sustainable pest management.

Phylogenetically, *C. agnata* belongs to the tribe Argyrogrammatini within the subfamily Plusiinae and is taxonomically related to other *Ctenoplusia* species, such as *C. limbirena*, *C. albostriata*, and *C. oxygramma*, which exhibit varying degrees of host plant specialization and geographic distribution.^7^ Understanding these evolutionary relationships and comparative host plant utilization patterns is essential for developing targeted pest management strategies across the subfamily. Despite the agricultural significance of this group, comprehensive genomic resources for *C. agnata* remain limited compared to some other Plusiinae species.

Chromosome‐level genome assemblies provide valuable data and tools for agricultural pest research, enabling the identification of genes associated with key biological processes including migration capacity, cold tolerance, insecticide resistance, and metabolic adaptations.^8^, ^9^ Although *C. agnata* is not currently considered a major insecticide‐resistant pest, understanding the genomic architecture of detoxification‐related gene families such as cytochrome P450 monooxygenases (CYPs), glutathione S‐transferases (GSTs), and carboxylesterases (CCEs) is critical for predicting potential adaptation to chemical stressors.^10^ Moreover, high‐quality genome assemblies facilitate the detection of structural variations that contribute to evolutionary divergence and ecological specialization.^11^

In this study, we present a high‐quality chromosome‐level genome assembly of *C. agnata*, generated using PacBio long‐read sequencing and Hi‐C scaffolding. Despite its agricultural significance, comprehensive genomic resources for *C. agnata* have remained limited compared to other Plusiinae members, impeding research on its gene content, structure, and function. This genome assembly fills the existing knowledge gap and provides a foundation for future studies on the molecular basis of the unique ecological traits of *C. agnata*. The chromosome‐level assembly enables comparative genomic analysis within Plusiinae, shedding light on evolutionary relationships and adaptation mechanisms in this agriculturally important group. Furthermore, high‐quality assemblies like this one are crucial for detecting structural variations that contribute to evolutionary divergence and ecological specialization, while offering insights into the genomic architecture of detoxification‐related gene families. This comprehensive genomic resource will enhance our ability to address pest management challenges and advance our understanding of the evolution of the Plusiinae subfamily and the broader Noctuidae family, ultimately facilitating more effective and sustainable pest control strategies.

## 2 MATERIALS AND METHODS

### 2.1 Insects

Larvae of *C. agnata* (Yanggu strain, looper_JK_23b) were collected from a soybean field in Yanggu, South Korea (latitude 38.17.16 N, longitude 128.26.18 E), on July 25, 2023 (Supporting Information, Fig. S1). The collected larvae were reared in the Insect Molecular Toxicology Laboratory (lab) at Kangwon National University under controlled lab conditions, simulating field‐relevant temperature, humidity, and photoperiod parameters as previously reported.^9^ The larvae were regularly monitored and provided with a consistent diet of fresh soybean leaves to support their development and ensure the reliability of experimental results.

For genomic DNA (gDNA) extraction, 5th‐instar male larva was selected, and DNA was isolated using the Quick‐DNA Miniprep Plus Kit (Zymo Research, Tustin, CA, USA), following a previously optimized protocol.^12^

For RNA sequencing, a mixed sample was used, consisting of more than 15 individuals pooled from different developmental stages (3rd and 5th larvae, pupae, and adults) to ensure a comprehensive representation of the transcriptome.

### 2.2 Genome sequencing and assembly

Purified pooled gDNA was sequenced using the PacBio High‐fidelity (HiFi) platform. HiFi reads were generated using circular consensus sequencing (CCS), ensuring high accuracy. CCS reads were processed using SMRT Link software (v10.1) with default settings, achieving at least 99% accuracy. Genome assembly was conducted using Hifiasm (v0.16.1‐r375) with default parameters.^13^ Genome completeness was assessed using BUSCO v5.0.0 with the insecta_odb10 dataset, revealing 98.8% completeness (1350/1367 complete BUSCOs).^14^

For scaffolding, a Pore‐C library was prepared following Ulahannan, Pendleton.^15^ The input library was prepared using the ONT SQK‐LSK114 kit, and sequencing was conducted on the PromethION platform (https://nanoporetech.com/products/sequence/promethion). Raw sequencing reads were base‐called using MinKNOW and converted to nucleotide sequences using Guppy (high‐accuracy model, 400 bps>Q8). Pore‐C reads were aligned to assembly contigs using Pore‐C‐Snakemake (v0.4.0), and scaffolding was performed using 3D‐DNA. Manual curation was conducted with Juicebox (Supporting Information, Fig. S2).

### 2.3 Gene annotation

For RNA‐seq‐based gene prediction, total RNA was extracted from *C. agnata* using the RNeasy Mini Kit (Qiagen, Hilden, Germany) following the manufacturer's instructions. RNA quality was assessed using an Agilent 2200 TapeStation (Agilent Technologies, Santa Clara, CA, USA), and RNA integrity was confirmed by 1% agarose gel electrophoresis. Bacterial contamination was removed using BBDuK tool with the bacterial RefSeq database.

To identify and annotate repeat elements within the genome, *de novo* analysis was performed using RepeatModeler (v2.0.1) (http://www.repeatmasker.org/RepeatModeler/).^16^ Tandem repeats were identified using Tandem Repeats Finder (v4.07b), while long terminal repeat (LTR) retrotransposons were characterized using LTR_Finder,^17^ LTR_Harvest,^18^ and LTR retriever.^19^ Identified repeat elements were subsequently analyzed and masked across the genome using RepeatMasker (v4.1.5) (http://www.repeatmasker.org) to prevent erroneous gene annotations due to repetitive sequences.^20^

Gene prediction was performed using BRAKER (v3.0.7), integrating evidence from transcriptomic and protein homology data. For transcriptomic data,^21^ RNA‐seq reads were aligned to the genome using HISAT2, while Iso‐Seq data underwent clustering and alignment through SMRT Link for long‐read transcript assembly.^22^

The annotation workflow utilized supporting evidence datasets from selected reference species based on their phylogenetic relatedness, high‐quality genome annotations, and availability of transcriptomic and protein datasets. The five reference species used were *C. includens* (Noctuidae, GCA_903961255.1), *T. ni* (Noctuidae, GCF_003590095.1), *Spodoptera litura* (Noctuidae, GCF_002706865.2), *S. exigua* (Noctuidae, GCA_015679615.1) and, *Drosophila melanogaster* (Drosophilidae, GCF_000001215.4). These species were chosen to provide a balance between close evolutionary relationships within Noctuidae and leveraging well‐annotated reference genomes such as *D. melanogaster* for functional insights. Additionally, RNA‐seq datasets from various developmental stages of *C. agnata* were incorporated into the gene prediction process (Supporting Information, Fig. S3A–C). Detailed software and parameter options are listed in Table 1.

**Table 1:** Used programs and options for gene prediction and annotation

Procedure	Program	Version	Options
Bacterial contig removal	BBDuK	38.87	‐k 23 ‐mcf 0.1
Repeat masking	RepeatModeler	2.0.5	Default
	LTR_Harvest	1.6.5	‐minlenltr 100‐maxlenltr 7000 ‐mintsd 4‐maxtsd 6 ‐motif TGCA‐motifmis 1 ‐similar 85‐vic 10‐seed 20
	LTR_Finder_parallel	1.2	Default
	LTR_retriever	2.9.9	Default
	RepeatMasker	4.1.6	Default
RNASeq	Trimmomatic	0.39	LEADING:3 TRAILING:3, SLIDINGWINDOW:4:20 MINLEN:50
	Hisat2	2.2.1	Default
IsoSeq	SMRTLink	13.0	(for HQ clustering) ‐ lima –isoseq ‐ isoseq refine ‐ isoseq cluster (alignment to genome) ‐ pbmm2 align ‐ isoseq collapse
Gene prediction	BRAKER	3.0.7	Default
	BUSCO	5.5.0	‐l insecta
Functional annotation	Diamond	2.1.9.163	max‐target‐seqs:20 evalue:1e‐5
	InterProScan	5.66–98.0	Default
	BLAST2GO CLI	1.5.1	Default
	BLAST2GO	5.2.5	Default
	KAAS (web tool)	‐	SBH method

Briefly, an evidence‐based gene model was constructed with GeneMakr‐ETP and gene models were refined using AUGUSTUS, a tool for predicting genes in genome sequences.^23^ TSEBRA was employed to integrate gene predictions from GeneMark‐ETP and AUGUSTUS to enhance accuracy, generating a comprehensive and high‐confidence gene set.^24^ To ensure repeat masking did not inadvertently remove genuine gene annotations, we intersected our repeat annotation with the gene annotation dataset to identify potential overlaps between gene exons and repetitive elements. Cases where gene exons overlapped with classified repeats were further inspected and manually curated to mitigate erroneous annotations.

The final set of predicted genes was functionally characterized using BLAST search against the NCBI nr database with the DIAMOND program,^25^ using max‐target‐seqs 20 and an e‐value cutoff 1e‐5. The description of the top hit was assigned to each gene function. The GO terms were assigned using BLAST2GO CLI (v.1.5.1) based on DIAMOND results. Protein domain annotation was performed using InterProScan (V5.66–98.0), and KEGG pathway analysis was conducted using the KAAS web tool^26^ with the single‐directional best‐hit (SBH) method.

### 2.4 Genome synteny

Genome synteny analysis was performed to assess the genomic collinearity and structural similarities between *C. agnata* and *C. includens*, to understand their evolutionary relationships and potential functional conservation. Given that *C. includens* has a well‐annotated reference genome (GCA_903961255.1), it was used as a reference to identify syntenic regions in *C. agnata*. MCScanX was employed to detect syntenic blocks and homologous gene pairs between the two species, facilitating the identification of conserved gene order and potential genome rearrangements.^27^ This analysis provides insights into chromosomal evolution, gene duplication events, and structural divergence between the species. The synteny results were visualized using Circos,^28^ generating a circular plot to illustrate chromosomal alignments and highlight conserved regions between *C. agnata* and *C. includens*.

### 2.5 Comparative genomics and GO analysis

For comparative genomics analysis, we employed a comprehensive approach to explore the evolutionary dynamics of *C. agnata* genes. OrthoFinder (https://github.com/davidemms/OrthoFinder) was first used to identify orthologous groups across selected species, enabling robust classification of gene families and their evolutionary relationships.^29^ The resulting orthologous groups were then analyzed using CAFÉ version 5.5.0, with default parameters to statistically detect gene family expansions and contractions based on phylogenetic models, providing insights into lineage‐specific genomic adaptations.^30^

To understand the biological significance of these evolutionary changes, we performed KEGG pathway analysis (https://www.kegg.jp) on the expanded and contracted gene families of *C. agnata*.^31^ The results were visualized using scatter plots to highlight significant pathway enrichments. Additionally, Gene Ontology (GO) (https://geneontology.org) enrichment analysis was conducted to elucidate the functional roles of genes in the expanded and contracted families, providing insights into their potential biological processes, molecular functions, and cellular components.^32^

### 2.6 Phylogenetic analysis and detoxification gene families in related species

A comprehensive phylogenetic analysis, together with gene family expansion and contraction analysis based on annotated open reading frames (ORFs) data sets, was conducted using OrthoVenn3 (https://orthovenn3.bioinfotoolkits.net). This complemented our OrthoFinder and CAFÉ analysis by allowing visualization and comparison of orthologous gene clusters among selected Noctuoidea species. The phylogenetic tree was constructed using the maximum likelihood method with the JTT + CAT evolutionary model. Gene family variation was assessed using CAFE5. *Lymantria dispar* was used as an outgroup to root the phylogenetic tree and provide evolutionary context.

To identify and analyze detoxification gene families, we examined five major detoxification‐related gene families: CYPs, ATP‐binding cassette transporters (ABCs), GSTs, UDP‐glucuronosyltransferase (UGTs), and carboxyl/cholinesterases (CCEs). Detoxification gene family members in *C. agnata* and other lepidopteran species were identified based on annotated genomic data and previous studies.^12^, ^17^, ^33^ Gene annotation and classification were further validated using InterProScan (v5.66–98.0, with adjusted parameters to improve domain detection) and Pfam domain searches. Homology‐based searches using BLASTP were performed against known detoxification genes from reference species. Specifically, CYPs were identified through BLASTP searches against the Arthropod Cytochrome P450 database (https://arthropodp450.eu/sequenceserver/).

For each detoxification gene family, incomplete gene models were filtered using specific criteria to ensure functional relevance. Only sequences containing key conserved domains (e.g., PF00067 for CYPs, PF00135 for CCEs) were retained. Sequences shorter than 150 amino acids and lacking conserved domains were excluded. Additional cross‐validation using BLAST2GO, KAAS, and manual inspection was performed to confirm domain presence and functional annotations. This domain‐based filtering approach refined the gene counts, ensuring a high‐confidence set of detoxification‐related genes, particularly in large and variable families such as CYPs and CCEs.

The chromosomal distribution of detoxification genes in *C. agnata* was determined using genome assembly data. Loci of these genes were visualized to assess their organization and potential diversification. A comparative analysis was conducted to evaluate interspecific variation in detoxification gene family sizes, particularly within Noctuidae and other lepidopteran taxa, to better understand the adaptive evolutionary patterns associated with detoxification capacity. All detailed annotation data supporting this study are available in Figshare at https://doi.org/10.6084/m9.figshare.28028162.v3.

## 3 RESULTS

### 3.1 Genome assembly and chromosome synteny

We generated a high‐quality, chromosomal‐level genome assembly for *C. agnata* (NCBI BioSample SAMN42356484) using PacBio Revio long‐read sequencing (approximately 235× coverage) and Pore‐C chromatin confirmation data. The final assembly (GCA_041146325.1) comprises 70 scaffolds, including 31 chromosomes and 81 unplaced contigs, totalling a 406.7 Mb genome. The scaffold N50 is 13.2 Mb, and the contig N50 (from pre‐scaffolding PacBio reads) is 13.1 Mb. The genome's GC content is 35.96%. The largest scaffold, Ca_Chr01, spans 23.66 Mb and consists of two contigs, while the smallest chromosomal scaffold, Ca_Chr31, measures 6.08 Mb (Table 2).

**Table 2:** Genome sequence information including genome assembly statistics

Assembly	Value
Genome size (Mb)	406.7
Number of scaffolds	70 (31 Chr + 39 contigs)
Length of scaffolds (bp)	406 728 298
Minimum length (bp)	12 618
Maximum length (bp)	23 663 150
Average length (bp)	5 810 404
Length of N50 scaffold (bp)	13 152 637
Length of N90 scaffold (bp)	9 315 113
GC content (%)	35.96
Repeat content (%)	28.57
BUSCO analysis (insecta)	
Total BUSCO groups searched	1367
Complete BUSCOs	1350 (98.8%)
Complete & single copy	1341 (98.1%)
Complete & duplicated	9 (0.7%)
Fragmented	6 (0.4%)
Missing	11 (0.8%)
Protein coding genes	
Predicted gene number	12 726
Functional annotated gene number	12 635 (99.28%)
Total length of genes (bp)	19 277 661
Smallest gene length (bp)	108
Largest gene length (bp)	29 223
Average gene length (bp)	1515
Number of annotated genes	
BLAST (DIAMOND)‐NCBI nr	12 633 (99.27%)
Protein domains (InterProScan)	10 267 (80.68%)
Gene Ontology (BLAST2GO)	9342 (73.41%)
KEGG pathway (KAAS webtools)	7079 (55.63%)
Repetitive elements	
DNA (bp)	4 848 351
LINE (bp)	18 265 598
LTR (bp)	4 133 466
SINE (bp)	1 685 598
Interspersed (bp)	113 041 640
Low complexity (bp)	559 170
Simple repeat (bp)	2 585 677
Unknown (bp)	71 156 849

Compared to other Pulsiinae species (Table 3), the *C. agnata* genome size (406.7 Mb) is comparable to *Autographa pulchrina* (426.2 Mb) and *Polychrysia moneta* (430.6 Mb), but slightly larger than *C. includens* (380 Mb) and *T. ni* (368.2 Mb). Its chromosome number (31) is consistent with most Plusiinae, except *T. ni* (28 chromosomes). The *C. agnata* genome encodes 12 726 genes—fewer than *T. ni* (17 482 genes) and *D. chrysitis* (18 320 genes)—but its higher sequencing depth (235×) ensures a robust assembly.

**Table 3:** Comparison of genomic information of 11 Plusiinae species registered in Genbank

Species	Genbank accession	Genome size (Mb)/No. of chromosome	No of genes	GC content (%)	Genome coverage (x)
*Abrostola tripartita*	GCA_905340225.1	381.0 (31)	13 011	37.0	54
*Autographa bractea*	GCA_964264225.1	412.4 (31)		35.5	50
*Autographa gamma*	GCA_905146925.1	373.1 (32)		35.5	36
*Autographa pulchrina*	GCA_905475315.1	426.2 (32)	15 972	35.5	47
*Chrysodeixis includens*	GCA_903961255.1	380.0 (31)	14 746	36.0	40
*Ctenoplusia agnata*	GCA_041146325.1	406.7 (31)	12 726	36.0	235
*Diachrysia chrysitis*	GCA_932294365.1	386.4 (31)	18 320	34.5	34
*Euchalcia variabilis*	GCA_964260715.1	452.0 (31)		35.5	35
*Plusia festucae*	GCA_950381575.1	422.5 (31)		35.5	66
*Polychrysia moneta*	GCA_964276815.1	430.6 (31)		35.0	89
*Trichoplusia ni*	GCF_003590095.1	368.2 (28)	17 482	35.5	600

Pore‐C sequencing generated 123 049 273 reads covering 37.3 Gb (Supporting Information, Fig. S4A), with an average read length of 3100 bp and a maximum of 657 689 bp. The N50 and N90 read lengths were 4055 bp and 1857 bp, respectively, confirming data suitability for scaffolding (Supporting Information, Fig. S4B). Integration of Pore‐C data improved assembly contiguity: the scaffold count was reduced from 81 to 70, with an N50 of 13.15 Mb and no gaps remaining in the final assembly (Supporting Information, Fig. S4C). The Proe‐C contact map (Supporting Information, Fig. S4D) shows a clear diagonal pattern, indicative of accurate chromosomal assignments, with minor off‐diagonal noise suggesting a small number of unresolved misassemblies.

**Figure 1:**
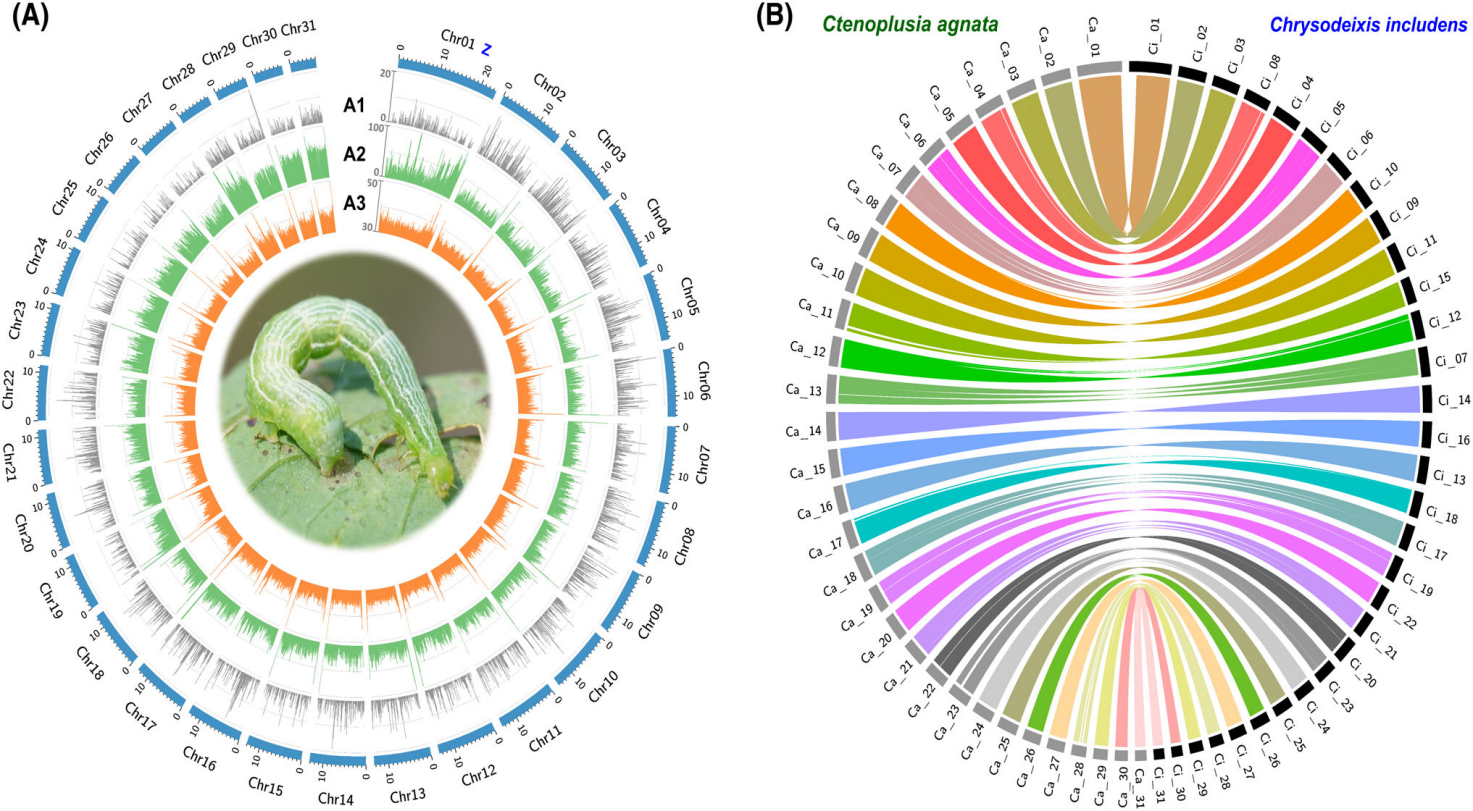
Circos plot of the *Helicoverpa assulta* genome (A) at the chromosomal level showed (A1) gene distribution, (A2) repeat distribution, and (A3) GC content. Chr01 is the sex chromosome, Z, and chromosomes 2 through 31 are all autosomes. Genome synteny (B) of *Ctenoplusia agnata* (Ca) and *Chrysodeixis includes* (Ci) was carried out using MCScanX and viewed with Circos (version 0.69). The chromosomes of *C. agnata* are shown in gray, and the chromosomes of *C. includes* is presented in black.

A circular genome visualization (Fig. 1A) highlights genome architecture, where chromosome 1 represents the Z chromosome and all others are autosomes. The outer ring depicts chromosomal lengths, while inner rings represent GC content, repeat density, and nucleotide diversity. The central image of the larva underscores the ecological importance of the species.

Comparative synteny analysis with *C. includens* (Fig. 1B) revealed largely conserved chromosomal structure with localized rearrangements—primarily inversions and translocations—on Ca_Chr07, Ca_Chr12, and Ca_Chr19. These may represent lineage‐specific adaptations with potential implications for gene regulation or chromosomal stability. Broader synteny comparisons with *T. ni* (Fig. S5) revealed greater genomic divergence and more extensive inter‐chromosomal rearrangements.

### 3.2 Genome annotation

The *C. agnata* genome assembly showed high completeness. Benchmarking Universal Single‐Copy Ortholog (BUSCO) analysis identifying 1350 (98.8%) of 1367 insect orthologs as complete, including 1341 (98.1%) single‐copy and 9 (0.7%) duplicated genes. Only 11 (0.8%) were missing, supporting the assembly's robustness. The genome spans 406.7 Mb across 70 scaffolds ranging from 12.6 Kb to 23.7 Mb. GC content is 35.6%, and repeat sequences make up 28.57% of the total genome (Table 2).

A total of 12 726 protein‐coding genes were predicted, with 12 635 (99.28%) receiving functional annotations. Gene length ranges from 108 bp to 29 233 bp, averaging 1515 bp. Short genes may represent truncated models or pseudogenes, requiring further validation. Among annotated genes, 12 633 (99.27%) had matches in the NCBI nr database *via* DIAMOND; 10 267 (80.68%) contained protein domains (InterProScan); 9342 (73.41%) were assigned Gene Ontology (GO) terms (BLAST2GO); and 7709 (55.63%) were mapped to KEGG pathways (KASS) (Table 2).

Gene number discrepancies across related species may reflect differences in annotation pipelines, sequencing depth, or biological variation such as gene gain/loss. Some unannotated genes may stem from lineage‐specific features or incomplete models, highlighting the need for further functional analysis.

### 3.3 Repetitive elements in *C. agnata* genome

Repeat annotation revealed that approximately 28.57% of the *C. agnata* genome consists of repetitive elements (Table 2). Among these, Long Interspersed Nuclear Elements (LINEs) were the most abundant, totaling 18.27 Mb, followed by Long Terminal Repeats (LTRs) at 4.13 Mb, DNA transposons at 4.85 Mb, and Short Interspersed Nuclear Elements (SINEs) at 1.69 Mb.

In addition to interspersed elements, simple sequence repeats (SSRs) and low‐complexity regions accounted for 2.59 and 0.56 Mb, respectively. A substantial portion of the repetitive landscape, approximately 71.16 Mb, was classified as unknown, highlighting the need for further characterization of these elements, which may include species‐specific or novel repeat families not yet represented in current databases.

### 3.4 Gene ontology

**Figure 2:**
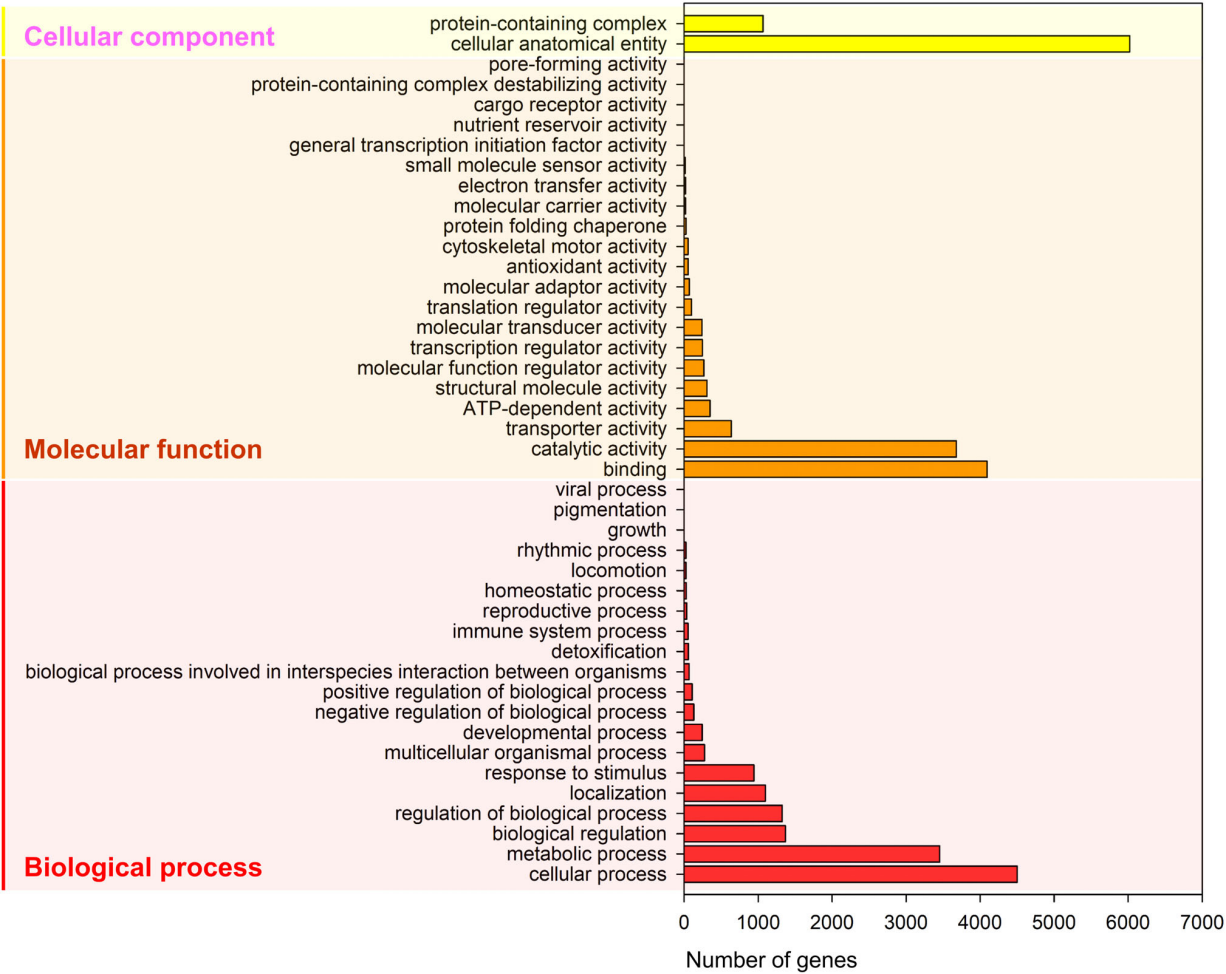
Blast2GO based gene ontology, GO‐slim distribution classified into three main categories of reference unigene: Cellular component, Molecular function, and Biological process. The detailed GO data is available in Figshare at https://doi.org/10.6084/m9.figshare.28028162.v3.

Gene Ontology (GO) analysis of the annotated unigenes in *C. agnata*, performed using Blast2GO and GO‐slim, offered a comprehensive functional overview across the three primary GO domains: cellular component, molecular function, and biological process. In the cellular component category, most unigenes were associated with cellular anatomical entities, protein‐containing complexes, while fewer were linked to membrane structures and pore‐related components. Within the molecular function domain, catalytic activity and binding were the predominant functions, reflecting the genome's involvement in diverse metabolic pathways and molecular interactions. Additional functions included transport activity, structural molecule activity, and ATP‐dependent activity. In the biological process domain, the majority of unigenes were involved in cellular and metabolic processes. Notably, genes related to detoxification, immune responses, and homeostasis were also identified, indicating potential mechanisms for environmental adaptations. Conversely, a smaller proportion of unigenes were linked to reproduction, locomotion, and rhythmic processes, suggesting ecological specialization (Fig. 2). Overall, this GO analysis underscores the functional diversity within the *C. agnata* genome and provides a valuable foundation for exploring its physiological and ecological adaptations.

### 3.5 Gene families expansion and contraction

We analyzed gene family evolution using OrthoVenn3 and CAFE5 to assess expansion and contraction events among *C. agnata*, *C. includens*, and *T. ni*.

**Figure 3:**
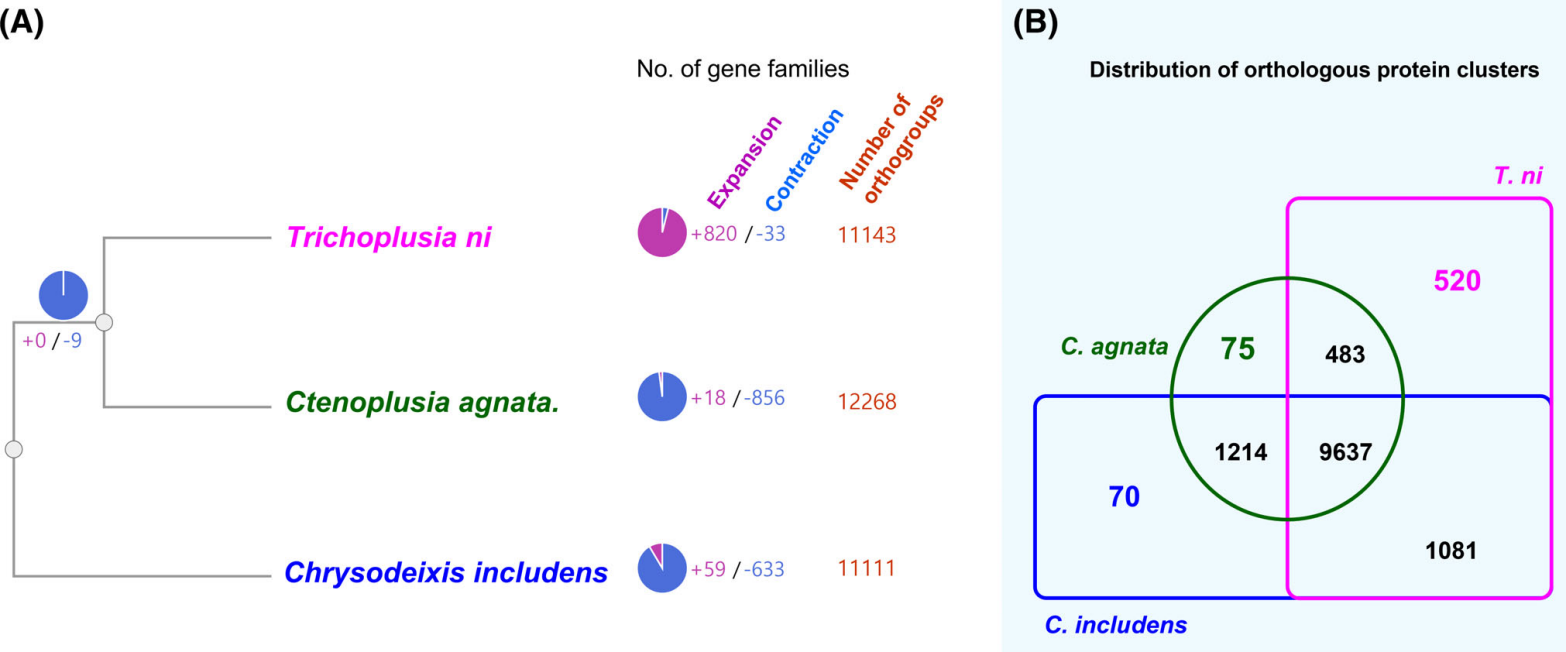
Analysis of orthologous protein clusters and gene families expansion and contraction using CAFE5 and OrthoVenn3. (A) Expansion and contraction analysis results. CAFE5 calculated the variation of the gene family, and the ultrametric tree showed a phylogenetic relationship among the three species based on the orthologous protein clusters (Number of orthogroups). Tree method: Maximum likelihood, Evolution Model: JTT + CAT. (B) The Edwards‐Venn diagram represents the distribution of orthologous protein clusters among *Ctenoplusia agnata*, *Chrysodeixis includes*, and *Trichoplusia ni*. The overlap areas indicate shared orthologous proteins, while the other areas indicate unique orthologous proteins.

The phylogenetic tree (Fig. 3A) indicates that *C. agnata* and *T. ni* share a more recent common ancestor than *C. includens*. Gene family evolution revealed marked differences: *T. ni* experienced extensive gene family expansion (+820), possibly driven by ecological adaptation. *C. agnata* showed modest expansions (+18) but substantial contraction (−856), suggesting genome streamlining or lineage‐specific losses.

An Edwards‐Venn diagram (Fig. 3B) identified 9637 shared gene clusters among the three species. *C. agnata* and *C. includens* share 1214 additional clusters, reflecting closer affinity. Species‐specific gene clusters were also observed: 520 unique to *T. ni*, 483 to *C. agnata*, and 1081 to *C. includens*, which may reflect divergent adaptations.

These results point to dynamic gene family turnover in Plusiinae, with varying evolutionary pressures contributing to their genomic diversity.

### 3.6 Genome family evolution and Detoxification gene analysis

**Figure 4:**
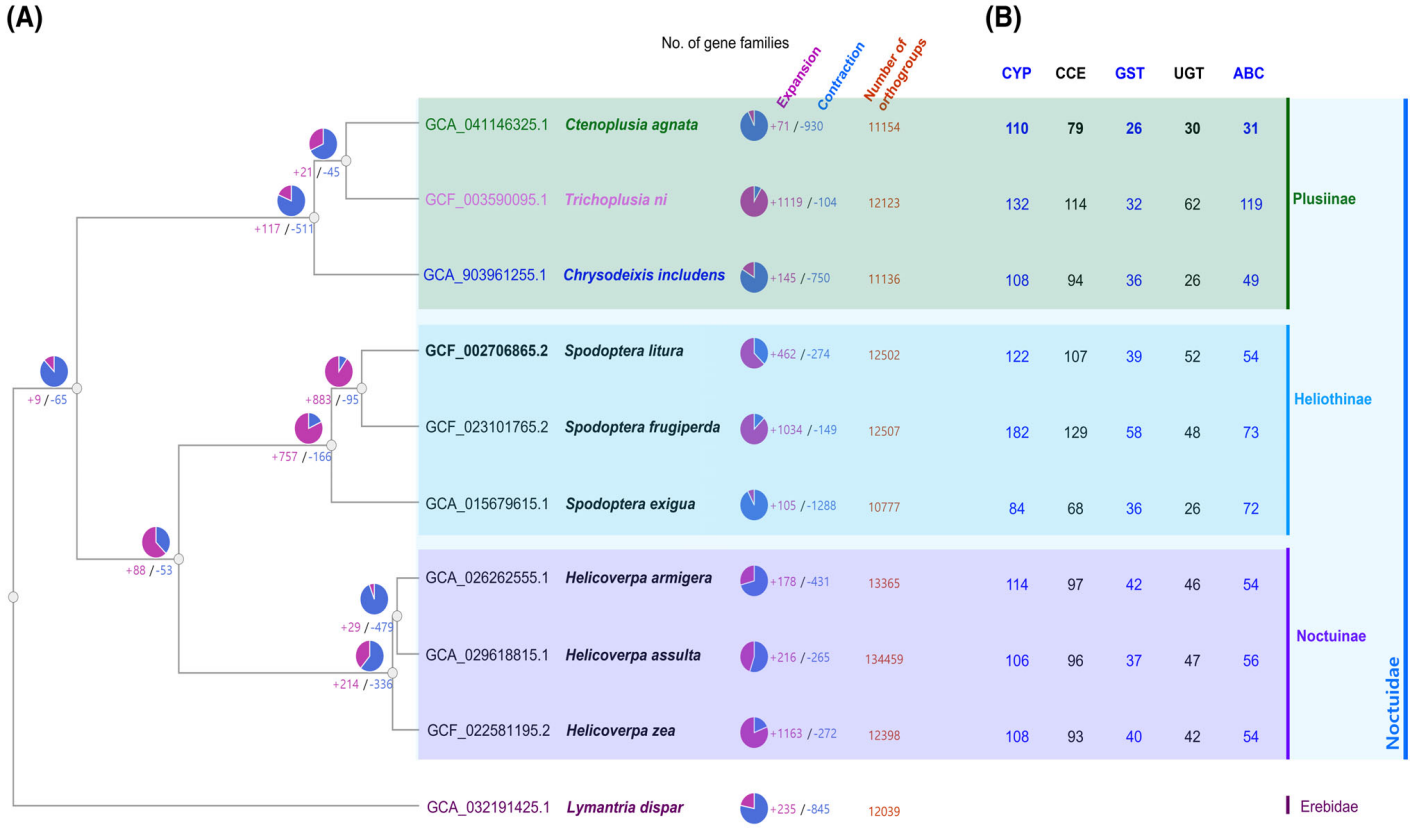
Comparison of representative Plusiinae with seven Noctuoidea genomes and of its number of five major detoxification genes. (A) A phylogenetic tree of these species was reconstructed based on genome wide open reading frame (ORF) data set using OrthoVenn3. OrthoVenn3 also visualizes evolutionary lineage and gene families' number of expansion and contraction. Tree method: maximum likelihood, Evolution model: JTT + CAT. The variation of gene family was calculated by CAFE5. (B) The number of detoxification gene members such as Cytochrome P450s (CYPs), carboxyl/cholinesterases (CCEs), glutathione S‐transferases (GSTs), UDPglucuronosyltransferases (UGTs), and ATP‐binding cassette transporters (ABCs) are presented per species. The number of detoxification enzyme genes was compiled through previously reported references and this study.^8^, ^13^, ^34^ The detailed annotation data is available in Figshare at https://doi.org/10.6084/m9.figshare.28028162.v4.

To explore broader evolutionary patterns in gene family dynamics, we compared expansions and contractions across various lepidopteran species, using *Lymantria dispar* as the outgroup to root the phylogenetic tree (Fig. 4A). Within the subfamily Plusiinae, *C. agnata* exhibited a net contraction (+71/−930), encompassing 11 154 orthogroups. Detoxification‐related gene families in *C. agnata* were represented by 110 CYPs, 79 CCEs, 26 GSTs, 30 UGTs, and 31 ABCs. In contrast, *T. ni* showed extensive expansion (+1119/−104) across 12 123 orthogroups, with higher counts in detoxification genes: 132 CYPs, 114 CCEs, 32 GSTs, 62 UGTs, and 119 ABCs. *C. includens* presented an intermediate pattern (+145/−750), with 11 136 orthogroups and 108 CYPs, 94 CCEs, 36 GSTs, 26 UGTs, and 49 ABCs.

In the Heliothinae, *S. frugiperda* exhibited the highest gene family expansion (+1034/−149, 12 507 orthogroups), with peak counts of 182 CYPs and 129 CCEs, alongside 58 GSTs, 48 UGTs, and 73 ABCs. *S. litura* also showed a net expansion (+462/−274), encompassing 12 502 orthogroups and detoxification gene counts of 122 CYPs, 107 CCEs, 39 GSTs, 52 UGTs, and 54 ABCs. In contrast, *S. exigua* showed a strong contraction (+105/−1288), with 10 777 orthogroups and lower detoxification gene numbers: 84 CYPs, 68 CCEs, 36 GSTs, 26 UGTs, and 72 ABCs. Among the Noctuinae, *Helicoverpa armigera*, *H. assulta*, and *H. zea* showed relatively balanced gene dynamics. *H. armigera* (+178/−431) and *H. zea* (+216/−265) had 13 365 and 13 459 orthogroups, respectively, with similar detoxification gene complements (CYPs: 114 and 106; CCEs: 97 and 96; GSTs: 42 and 37; UGTs: 46 and 47; ABCs: 54 and 56). *H. zea* displayed the largest expansion in this group (+1163/−272), with 12 398 orthogroups and detoxification gene counts of 108 CYPs, 93 CCEs, 40 GSTs, 42 UGTs, and 54 ABCs. The outgroup, *L. dispar*, included 12 039 orthogroups, with 235 expanded and 845 contracted gene families.

**Figure 5:**
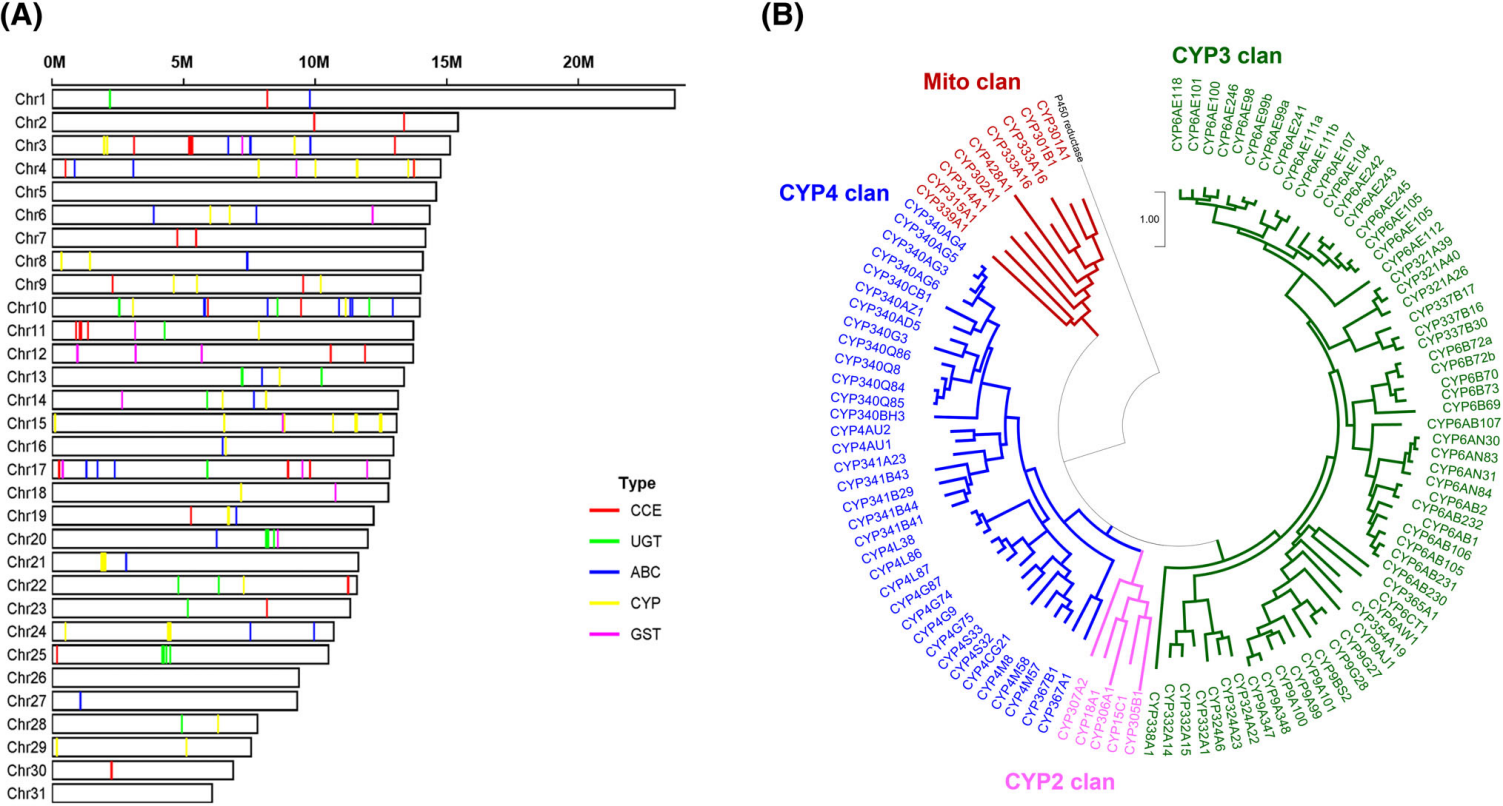
Chromosomal distribution and phylogenetic relationships of Cytochrome P450s (CYPs) in *Ctenoplusia agnata*. (A) Chromosomal mapping of major detoxification gene families across the 31 chromosomes of *C. agnata*. Horizontal bars represent individual chromosomes (Chr1–Chr31) with relative lengths indicated by the scale bar (0–20 M). Colored vertical lines denote the locations of five key detoxification gene families: CCE (red), UGT (green), ABC (blue), CYP (yellow), GST (pink). (B) Phylogenetic analysis of the CYP gene family in C. agnata. The circular dendrogram illustrates the evolutionary relationships among CYP genes, grouped into four clans: mitochondrial (red), clan 2 (pink), clan 3 (green), and clan 4 (blue). Terminal branches represent individual CYP genes, with the scale bar (1.00) indicating genetic distance.

To further investigate the genomic architecture and evolutionary diversification of detoxification‐related gene families, we examined their chromosomal distribution and phylogenetic relationships (Fig. 5). In panel A, the five major detoxification gene families—CYPs, GSTs, CCEs, UGTs, and ABCs—are shown to be broadly distributed across nearly all 31 chromosomes in *C. agnata*, rather than being restricted to specific loci. This dispersed genomic dispersion suggests that these gene families may have evolved independently, potentially supporting diverse regulatory mechanisms and functional specialization. Interestingly, certain chromosomes harbored higher densities of specific detoxification genes, hinting at potential functional hotspots or past gene duplication events.

Panel B presents a phylogenetic reconstruction of the CYP gene family in *C. agnata*, which clusters into four major clans: mitochondrial, clan 2, clan 3, and clan 4. The circular phylogenetic tree highlights both deep ancestral splits and more recent duplication events within *C. agnata*. Notably, clans 3 and 4 appear particularly expanded within this species, suggesting that these subfamilies play a central role in ecological adaptation. Their diversification could enhance substrate flexibility and support the detoxification of a broad range of xenobiotics, including allelochemicals and synthetic insecticides. These patterns highlight how internal gene family expansions may contribute to the adaptive potential of *C. agnata* in chemically complex environments.

## 4 DISCUSSION

Chromosome‐level genome assemblies provide critical insights into the genomic structure, evolutionary biology, and ecological adaptations.^35^ In this study, we present a high‐quality chromosome‐level genome assembly of *C. agnata*, marking a significant step forward in understanding genome organization and pest biology within the Plusiinae subfamily. The assembly spans 406.7 Mb across 31 chromosomes, within an N50 of 13.2 Mb and 98.8% BUSCO completeness, providing a robust foundation for comparative genomics and functional studies (Table 2). Our findings underscore the agricultural importance of *C. agnata* and its close relatives, *T. ni* and *C. includens*, both of which are notorious agricultural pests with extensive crop‐damaging potential.^36^

The assembly was achieved using advanced sequencing technologies, including PacBio Revio long‐read sequencing and Pore‐C scaffolding, enabling exceptional genome continuity and accuracy.^37^ These methodologies were instrumental in resolving complex genomic regions and scaffolding contigs into chromosome‐scale structures.^38^, ^39^ The resulting genome is among the most complete in lepidopteran assemblies, surpassing previously published genomes of other Noctuidae species, such as *Helicoverpa assulta*.^9^, ^40^ Similar efforts in insects such as *Dysgonia stuposa* have demonstrated how high‐quality genome assemblies can enhance our understanding of pest biology and guide pest management strategies.^41^

Previous studies investigating Noctuidae evolutionary relationships primarily used a limited number of molecular markers. For example, Sinha and Shashank^42^ employed mitochondrial (*COI*) and nuclear (*RPS5*) genes to explore phylogenetic relationships within Indian Plusiinae. Building on this foundation, our whole‐genome analysis employed a set of orthologous single‐copy genes, aligning their amino acid sequences to establish a more comprehensive framework for understanding the evolution of *C. agnata* and related taxa. This aligns with recent advances in lepidopteran systematics, such as the transcriptomic phylogenomic study of Lepidoptera by Kawahara, Plotkin,^43^ which found that Lepidoptera diversified around 300 million years ago, revealing the ancient origin of the group that may have shaped modern pest species like *C. agnata*. This study, coupled with Li, Breinholt^44^ findings, further contextualizes the divergence of key pest species like *C. agnata* and *T. ni* and highlights the evolutionary depth that informs the genetic diversity observed in pest adaptation.

Synteny analysis between *C. agnata* and *C. includens* revealed extensive genomic collinearity, indicating conserved chromosomal architecture with minimal rearrangements. In contrast, comparisons with *T. ni* showed greater structural divergence, pointing to lineage‐specific genomic evolution within Noctuidae (Fig. 3 and Supporting Information, Fig. S5). These results align with previous lepidopteran studies that highlight the significance of conserved genomic regions in shaping adaptive responses.^40^, ^41^

Although *C. agnata* predicted gene count was lower than those of related species, this may reflect differences in annotation pipelines, genome assembly quality, or lineage‐specific gene gain/loss. Additionally, under‐annotated regions or species‐specific genome streamlining may have contributed. These possibilities merit further exploration through transcriptomic or functional genomic studies.

Our analysis prioritized detoxification‐related gene families as key focus, due to their central role in pest adaptation and resistance to insecticides.^45^, ^46^ The CYP family, in particular, mediates phase I detoxification through xenobiotic oxidation and exhibits considerable interspecies variability.^47^ We identified 110 CYP genes in *C. agnata*, a lower number compared to *S. frugiperda* (182) (Fig. 4A), which is known for its robust detoxification potential.^34^, ^45^, ^48^ Phylogenetic analysis of the CYP gene family (Fig. 5B) showed a pronounced expansion of the CYP clan, which is particularly important in the detoxification of plant allelochemicals and synthetic insecticides.^49^ This expansion mirrors patterns observed in species like *S. litura* ^50^ and *H. armigera*,^51^ where members of the CYP6 and CYP9 subfamilies have been directly implicated in insecticide resistance *via* overexpression or adaptive mutations.^52^ Conversely, the relatively fewer CYP2 and Mito clan genes in *C. agnata* suggest reduced emphasis on core physiological roles and a shift toward xenobiotic metabolism specialization.

The GSTs, which catalyze phase II detoxification *via* conjugation, are another critical family with variable representation across species.^53^ *C. agnata* encodes 26 GST genes, compared to 58 in *S. frugiperda* (Fig. 4B). The expanded GST profiles in Noctuids underscore their high metabolic adaptability to insecticidal stressors.^34^, ^54^, ^55^

ABCs, which mediate xenobiotic efflux, further illustrate detoxification capabilities in pests.^56^ *C. agnata* contains 31 ABC genes, whereas *S. frugiperda* and *T. ni* 73 and 119, respectively (Fig. 4A). The more extensive ABC repertoire in other Noctuids supports their ability to manage xenobiotic loads and develop resistance,^57^, ^58^, ^59^ in line with broader findings on detoxification gene diversity contributing to pest resilience.^60^, ^61^

These genomic differences suggest that while *T. ni* exhibits a more expansive detoxification capacity—contributing to its widespread pest status—*C. agnata* demonstrates a more moderate profile. This may apply as a narrower but still effective metabolic toolkit, sufficient for its current pest status, yet possibly limiting its potential for broad‐spectrum resistance. In contrast to more chemically resilient *T. ni* and *H. armigera*, *C. agnata* may rely on alternative ecological or behavioral mechanisms for adaptation.

The chromosomal mapping of detoxification genes in *C. agnata* (Fig. 5A) revealed a dispersed distribution, rather than gene clustering, across most chromosomes. This dispersedgenomic architecture implies independent gene family evolution and possibly diverse regulatory mechanisms, contrasting with clustered gene arrangements observed in other insect species.

Furthermore, when comparing detoxification gene counts with related species, *C. agnata* showed similar patterns to *C. includens* (108 CYP, 94 CCE, 36 GST, 26 UGT, and 49 ABC genes), but lower numbers than *T. ni* (132 CYP, 114 CCE, 32 GST, 62 UGT, and 119 ABC). These differences likely reflect distinct adaptive strategies within Plusiinae and could inform tailored pest control approaches.^9^, ^40^

In summary, this chromosome‐level genome assembly provides a comprehensive platform for advancing research into *C. agnata* genome structure, detoxification capacity, and pest adaptation. While traits such as long‐distance migration and cold tolerance are ecologically relevant, they were beyond the primary scope of this study, which focused on detoxification gene families. However, the availability of a high‐quality reference genome now enables future investigations into such traits through comparative genomics, transcriptomics, and functional assays. This work lays the groundwork for precision pest management strategies targeting detoxification pathways and offers a valuable genomic reference for broader studies in Plusiinae biology.

## 5 CONCLUSION

We present a high‐quality, chromosome‐level genome assembly for *C. agnata*, spanning 406.7 Mb across 31 chromosomes with 98.8% BUSCO completeness. Generated using PacBio Revio long‐read sequencing and Pore‐C scaffolding, this assembly exhibits exceptional continuity and completeness, representing one of the most comprehensive genomic resources available for the subfamily Plusiinae. Comparative synteny analysis revealed substantial chromosomal conservation with *C. includens*, disrupted by only localized rearrangements on Ca_Chr07, Ca_Chr12, and Ca_Chr19, while broader comparisons with *T. ni* highlighted greater structural divergence, underscoring distinct evolutionary trajectories. Gene family evolution analysis indicated extensive contraction (−1064 families) and limited expansion (+26 families) in *C. agnata*, contrasting sharply with the genomic plasticity observed in *T. ni* (+1123/−56). These patterns may reflect lineage‐specific adaptations linked to host plant interactions and environmental pressures. Repeat content analysis showed that 28.57% of the *C. agnata* genome comprises repetitive elements, with LINEs as the dominant class, consistent with lepidopteran genome architecture. Our annotation revealed 12 726 protein‐coding genes, with 99.28% functionally annotated. GO analysis emphasized roles in metabolism, detoxification, immune responses, and cellular responses, suggesting a versatile functional repertoire potentially supporting ecological adaptation. Notably, *C. agnata* possesses a moderate complement of detoxification‐related genes, including 110 CYPs, 79 CCEs, 26 GSTs, 30 UGTs, and 31 ABCs. Chromosomal mapping revealed a widespread genomic distribution of detoxification genes, with no evidence of clustering, implying independent evolutionary origins and possible regulatory diversification. Phylogenetic analysis of CYPs revealed enrichment in the CYP3 and CYP4 clans—especially the former—suggesting enhanced capacity for detoxifying xenobiotics, such as allelochemicals and synthetic insecticides. Compared to related species, *C. agnata* exhibits a detoxification gene profile that is slightly less extensive than *C. includens* and substantially reduced relative to highly polyphagous and insecticide‐resistant species like *T. ni* and *S. frugiperda*. These differences likely reflect varying metabolic capacities and may influence the ecological range and resistance potential of *C. agnata*. This genome assembly provides a valuable foundation for studying the molecular basis of adaptation and resistance in *C. agnata* and other noctuid pests. It also offers practical insights for developing targeted and sustainable pest management strategies. Future research should prioritize the functional validation of key detoxification genes to better understand the mechanistic underpinnings of insecticide metabolism and resistance evolution in this economically significant species.

## AUTHOR CONTRIBUTIONS

J.K. designed the research for the whole paper; J.K., H.F., and M.K. coordinated the project; J.K., H.F., and M.K prepared the original draft; J.K and M.K. revised the paper; J.K prepared the samples, analyzed the data, and annotated the genomes.

## CONFLICT OF INTEREST

The authors declare no competing interests.

## Supporting information


**Data S1.** Supporting Information.

## Data Availability

The data that support the findings of this study are openly available in figshare at https://doi.org/10.6084/m9.figshare.28028162.v4, reference number 28028162.
