# Supplementary material for: Chromosome‐level genome assembly of Ctenoplusia agnata and its potential application in Plusiinae pest management
Source: Pest Manag Sci. 2025 Jun 5;81(9):5961–72. doi: 10.1002/ps.8949 (PMC12332114; doi:10.1002/ps.8949)
Supplement: Supplementary file 1 — Data S1. Supporting Information. [file PS-81-5961-s001.docx]

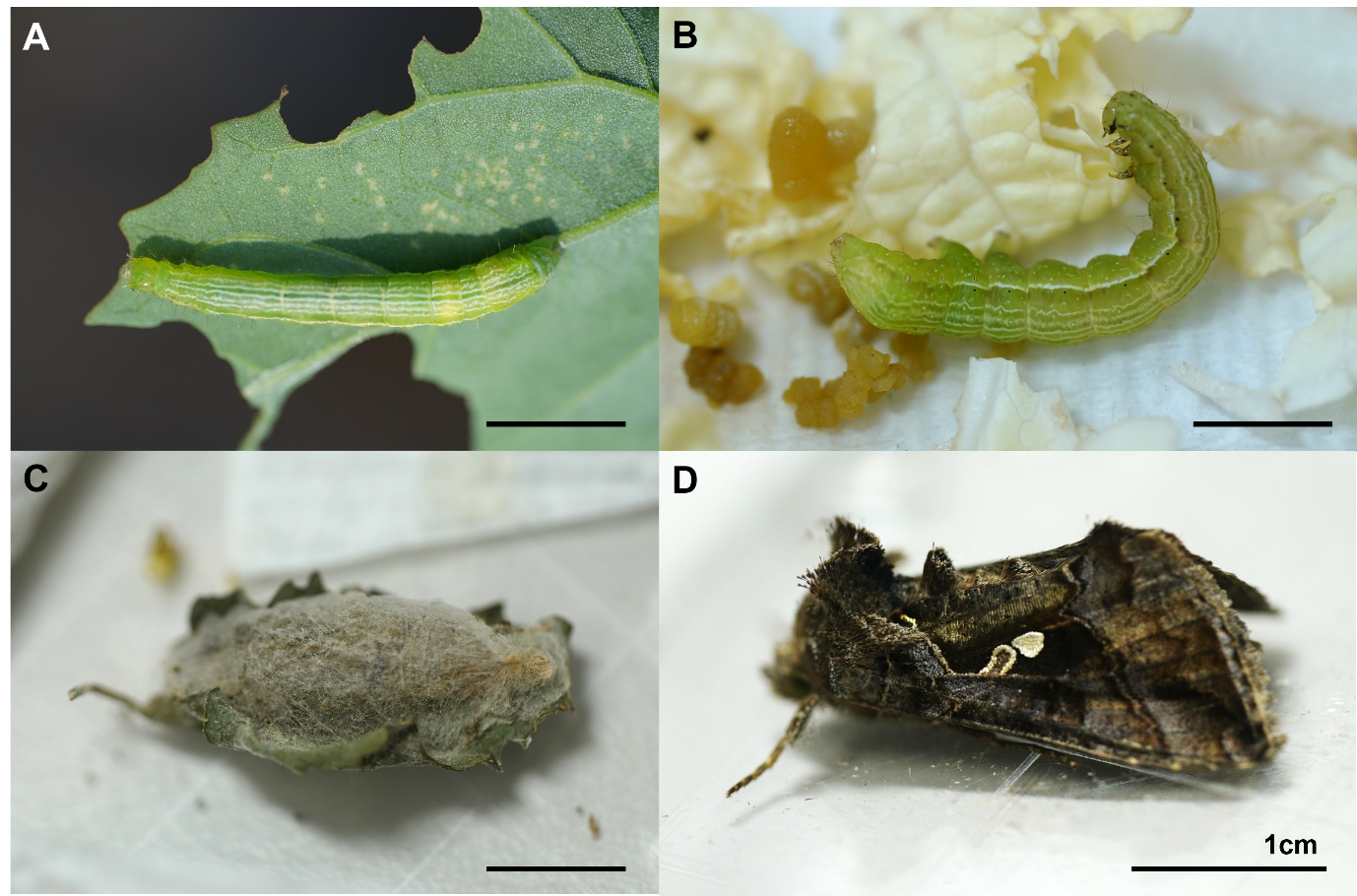


**Figure S1.** Photographs of larva (A and B), pupa (C), and adult (D) stages of *Ctenoplusia agnata* which used for genome analysis. Feeding of 5th and 6th instar larvae (A) Goosefoot, *Chenopodium album* (B) Kimchi cabbage, *Brassica rapa*.


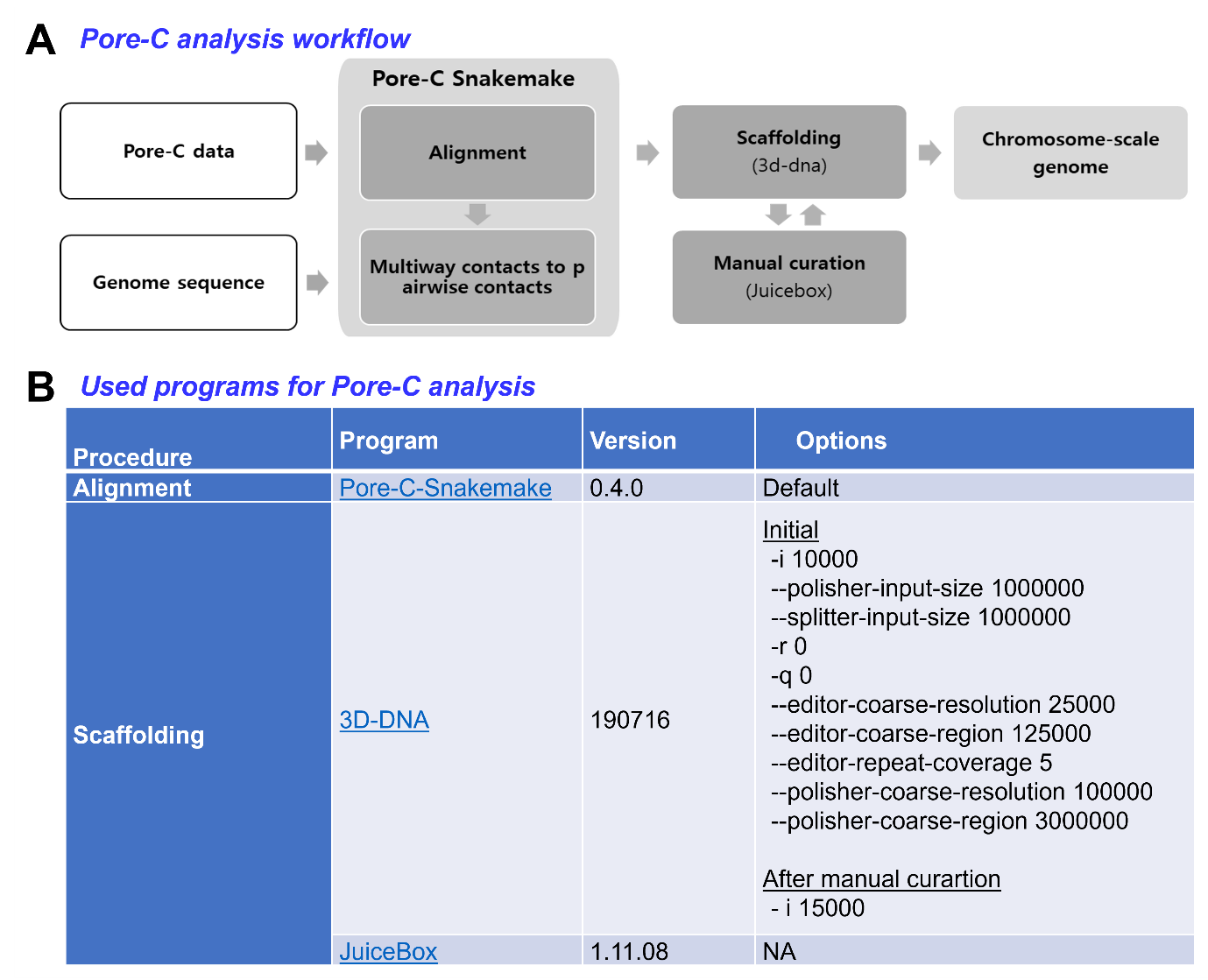


**Figure S2.** Summarized workflow of the pore-c analysis (A) and detailed information of the used program (B). The pore-c analysis is primarily divided into alignment and scaffolding stages. The Pore-C and genome sequencing data were aligned using the Pore-C Snakemake program and then scaffolded using the 3D-DNA program. Finally, the JuiceBox program was used for manual curation to assemble the chromosome-level genome.


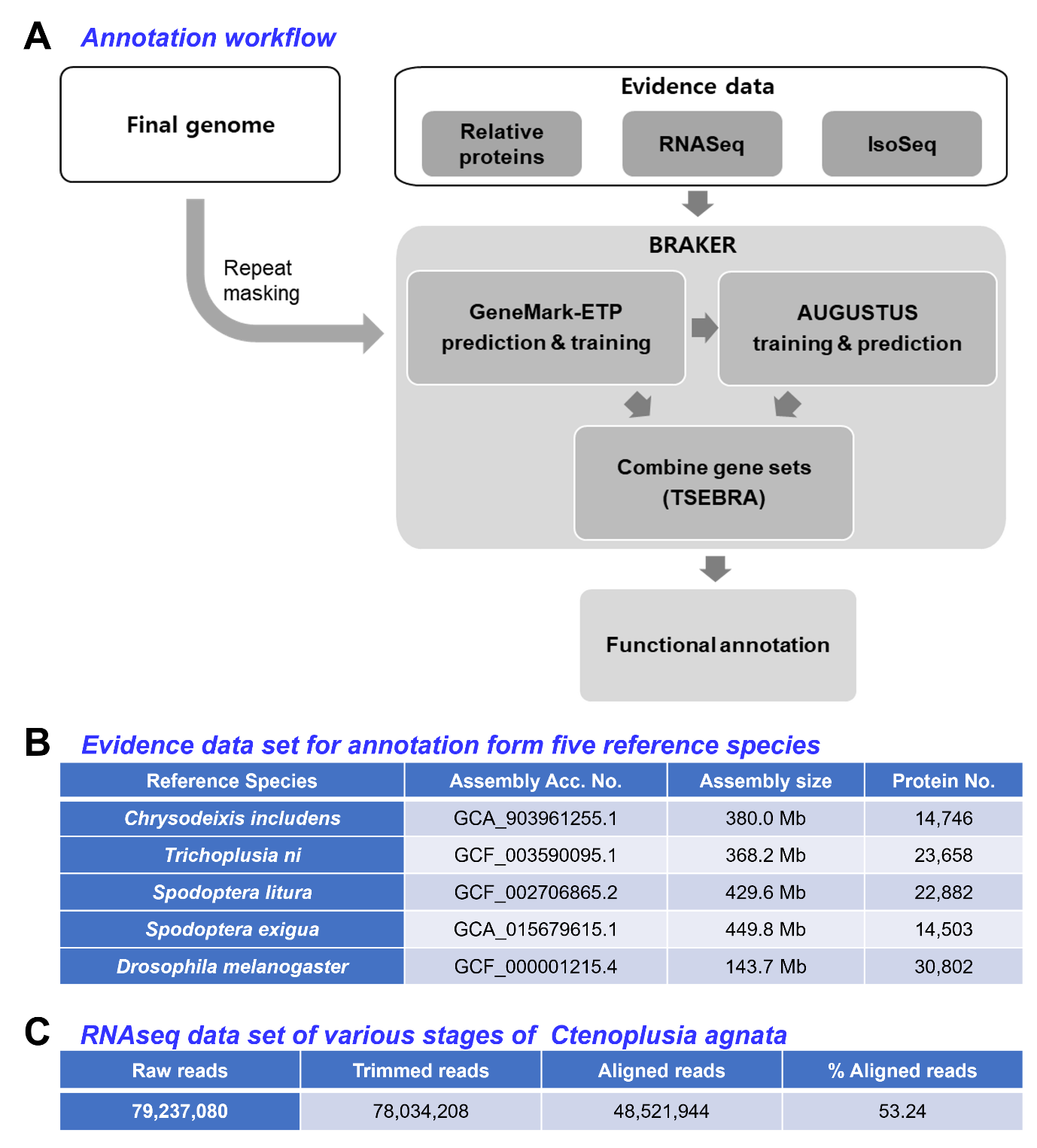


**Figure S3.** Summary of annotation workflow and details. (A) Annotation was divided into the following three steps. 1) Repeat masking: We used RepeatModeler to identify de novo repeat families and LTR_Finder, LTRharvest, and LTR_retriever to identify LTR retrotransposon families additionally. 2) Evidence data preparation: We used the protein sequence information of five species, including closely related species, RNAseq results, and Isoseq results as evidence data. 3) Gene prediction by BRAKER: Detailed program information and details are summarized in Materials and Methods and Table 1. (B) Evidence data set for annotation from five reference species. (C) RNAseq data set of various stages of *C. agnata*.

**
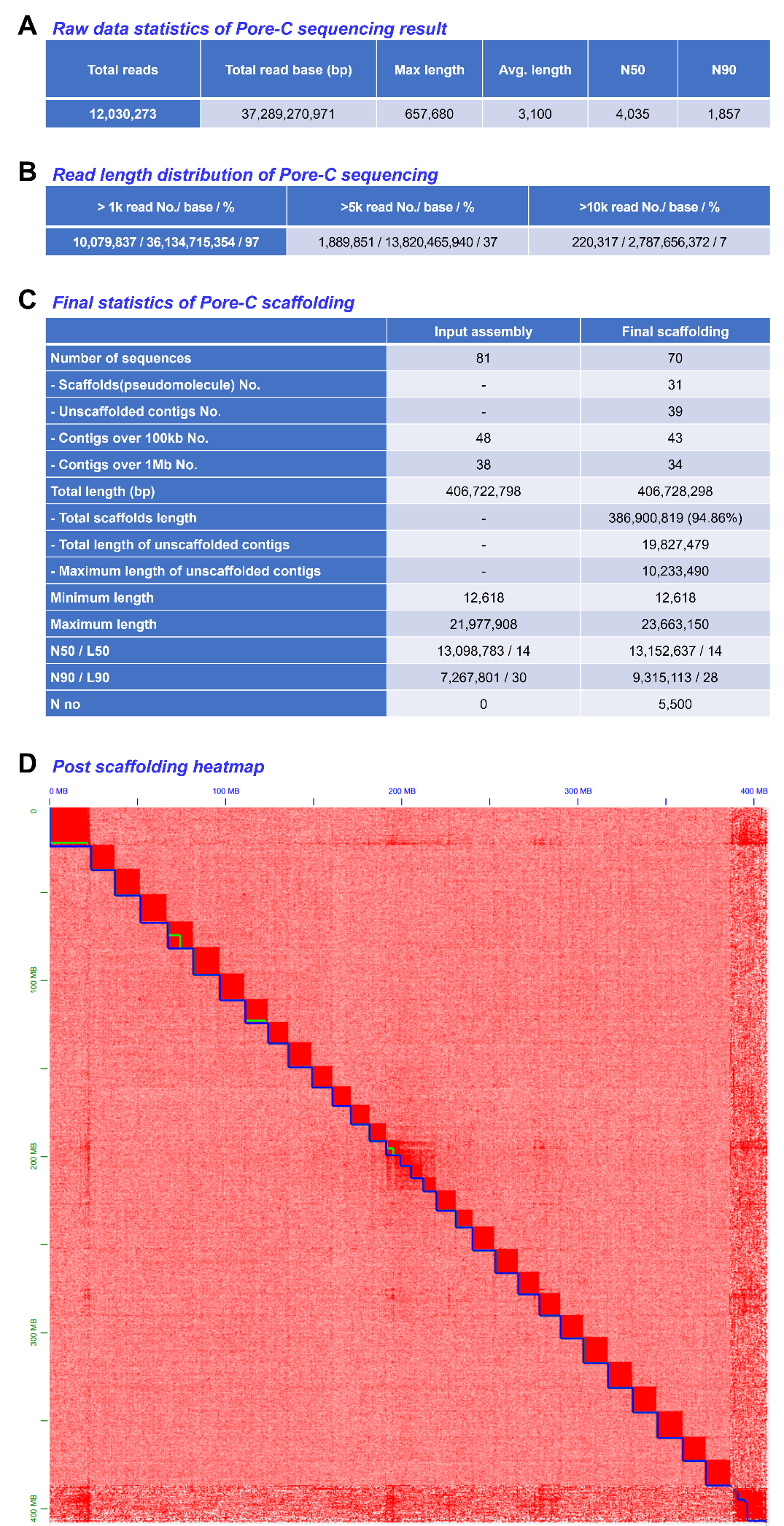
**

**Figure S4.** Summary of Pore-C results. (A) Raw data statistics of Pore-C sequencing result. (B) Read length distribution of Pore-C sequencing. (C) Final statistics of Pore-C scaffolding. (D) Post scaffolding heatmap: Review Hi-C signals with the Juicebox program to perform manual curation and generate pairwise contact heatmap. The detailed Pore-C data is openly available in Figshare at https://doi.org/10.6084/m9.figshare.28028162.v1


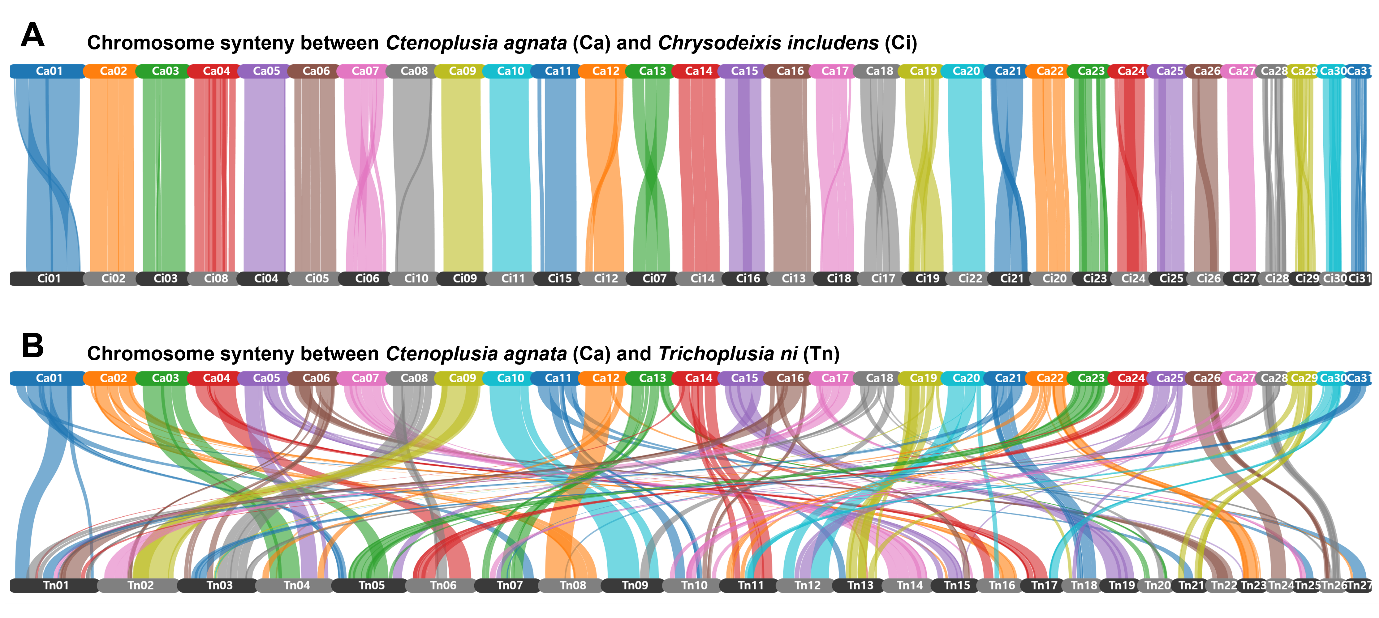


**Figure S5.** Genome synteny (A) between of Ctenoplusia agnata (Ca) and Chrysodeixis includens (Ci), (B) *C. agnata* (Ca) and *Trichoplusia ni* (Tn). Synteny analysis was carried out using MCScanX and visualized with SynVisio.
